# Ridge regression and deep learning models for genome-wide selection of complex traits in New Mexican Chile peppers

**DOI:** 10.1186/s12863-023-01179-6

**Published:** 2023-12-18

**Authors:** Dennis N. Lozada, Karansher Singh Sandhu, Madhav Bhatta

**Affiliations:** 1 Department of Plant and Environmental Sciences, New Mexico State University, Las Cruces, NM 88003 USA; 2 Chile Pepper Institute, New Mexico State University, Las Cruces, NM 88003 USA; 3 Bayer Crop Science, Chesterfield, MO 63017 USA

**Keywords:** *Capsicum* spp., Genomic prediction, Genomic estimated breeding values, Linkage disequilibrium, Machine learning, Plant morphology, Single nucleotide polymorphisms, Yield

## Abstract

**Background:**

Genomewide prediction estimates the genomic breeding values of selection candidates which can be utilized for population improvement and cultivar development. Ridge regression and deep learning-based selection models were implemented for yield and agronomic traits of 204 chile pepper genotypes evaluated in multi-environment trials in New Mexico, USA.

**Results:**

Accuracy of prediction differed across different models under ten-fold cross-validations, where high prediction accuracy was observed for highly heritable traits such as plant height and plant width. No model was superior across traits using 14,922 SNP markers for genomewide selection. Bayesian ridge regression had the highest average accuracy for first pod date (0.77) and total yield per plant (0.33). Multilayer perceptron (MLP) was the most superior for flowering time (0.76) and plant height (0.73), whereas the genomic BLUP model had the highest accuracy for plant width (0.62). Using a subset of 7,690 SNP loci resulting from grouping markers based on linkage disequilibrium coefficients resulted in improved accuracy for first pod date, ten pod weight, and total yield per plant, even under a relatively small training population size for MLP and random forest models. Genomic and ridge regression BLUP models were sufficient for optimal prediction accuracies for small training population size. Combining phenotypic selection and genomewide selection resulted in improved selection response for yield-related traits, indicating that integrated approaches can result in improved gains achieved through selection.

**Conclusions:**

Accuracy values for ridge regression and deep learning prediction models demonstrate the potential of implementing genomewide selection for genetic improvement in chile pepper breeding programs. Ultimately, a large training data is relevant for improved genomic selection accuracy for the deep learning models.

**Supplementary Information:**

The online version contains supplementary material available at 10.1186/s12863-023-01179-6.

## Introduction

Advances in next-generation sequencing technologies in recent years have revolutionized plant breeding for genetic improvement. The availability of whole genome sequences for major staple crops and specialty vegetables has driven molecular marker discovery and the implementation of marker-assisted selection (MAS) in breeding programs and have catalyzed the development of genetically improved crops [1]. Chile pepper (*Capsicum* spp.) is a widely used spice in many areas of the world and is a major cultural and economic crop in the state of New Mexico, USA. The use of novel genomics-assisted breeding approaches can drive genetic improvement and increase the total production in chile pepper growing areas. Integrating different ‘-omics’ tools will be a key to develop genetically improved cultivars of chile peppers for growers and consumers [2].

The identification of genotype-phenotype associations is a key step for MAS [3]. A major approach to determine significant marker-trait associations is genomewide association study (GWAS), which implements a linkage disequilibrium (LD)-based mapping approach to discover quantitative trait loci (QTL) in natural populations [4, 5]. Nonetheless, GWAS can suffer from the presence of population structure, effect of “missing heritability”, and detection of only major effect QTLs, hence imposes several disadvantages [6–8]. Another MAS tool, genomic prediction or genomewide selection, can complement GWAS through the estimation of genomic breeding values of selection candidates.

First proposed by Meuwissen et al. [9], genomic prediction uses genomewide marker data to predict the breeding values (genomic estimated breeding values, GEBVs) of selection candidates by using phenotypic and genotype information from a training population and genotype information from a test (validation) population [10]. In contrast to GWAS, genomic prediction estimates effects of markers across the whole genome based on the prediction model developed in the training population and eliminates the need to identify individual significant associations [11]. The correlation between the observed phenotypes and the GEBVs represent the accuracy of genomic prediction and is affected by several factors including the size of the training population, marker number, genetic relatedness between the training and validation populations, trait heritability, and the presence of fixed effects in the selection model, among others, across different crops [12–18].

Only a few studies have applied genomic prediction to estimate accuracy of quantitative traits in chile peppers. Hong et al. [19] performed predictions for fruit traits in 351 accessions of a *Capsicum* core collection, where it was observed that Reproducing Kernel Hilbert Space models have the highest accuracies of 0.75, 0.73, 0.84, 0.83, and 0.82 for fruit length, shape, width, weight, and thickness of pericarp, respectively. In another study, Tong et al. [8] combined a high-throughput phenotyping tool, Tomato Analyzer [20, 21], with genomic prediction to characterize a population of chile peppers from the Balkan region of Europe based on morphometric and colorimetric descriptors and observed a predictability value of 0.89 for fruit weight. Improvement on the genomic prediction accuracies from up to 10% was observed when markers were included as fixed effects in ridge regression and Kernel-based prediction models for capsaicinoid content [22].

While various BLUP-based and Bayesian models have been widely used to predict complex traits in crops, some of them only model the additive component of the variance [23, 24]. Machine learning and deep learning-based models can include the non-genetic effects with appropriate parameterization where the whole genetic merit can be predicted without the need of partitioning these non-genetic effects [25]. Deep learning is a sub-branch of machine learning which consists of a heterogeneous collection of machine learning algorithms that excelled at many prediction tasks and is currently an active area of research in most of the science fields [26]. These models use a combination of neurons and layers, where data is transformed multiple times to find the best fit. Implementation of deep learning models is straightforward with free access to the ‘Keras’ and ‘Scikit’ libraries; however, optimum model performance depends upon the hyperparameter used, which is not a trivial task, and requires huge computational resources and iterations [27]. Sandhu et al. [28] and Sirsat et al. [29] have shown that machine and deep learning-based models result in higher prediction accuracies for complex traits in wheat using different feature selection parameters and cross-validation approaches.

To date, genomic prediction studies in chile peppers, particularly on yield related and plant phenology and morphology-traits remain limited, and hence there is a need to explore different prediction approaches to characterize these quantitative traits in chile pepper. The objectives of the current study were to: (1) determine the accuracy of genomic prediction for yield and agronomic traits including plant morphology and phenology-related traits in *Capsicum* spp. using various ridge regression and deep learning models; (2) identify the effects of using marker subsets on the accuracy of genomic prediction; and (3) calculate the selection response of various selection strategies for yield and related traits. Six different models were used for genomewide selection: Bayesian ridge regression (BRR), genomic best linear unbiased prediction (GBLUP), ridge regression best linear unbiased prediction (RRBLUP); and deep learning models, viz., convolutional neural network (CNN), multilayer perceptron (MLP), and random forest (RF) for a tested genotypes in tested environment genomic prediction approach.

## Results

### Accuracy of genomic prediction and genomic heritability

Mean genomic prediction accuracy varied across traits and models ranging between 0.02 (green yield per plant; RRBLUP) and 0.77 (first pod date; BRR) using whole marker data (14,922 SNP markers; Additional file 1, Tables S1 and S2). Average accuracies across the six models were highest for the phenology-related traits, first pod date and flowering time (*r =* 0.76), followed by plant height (0.71) and ten pod weight (0.69) using whole marker dataset for genomic prediction (Additional file 2, Tables S1-S8). In contrast, the traits with the least average prediction accuracies were the yield and yield components, viz., mature green yield (0.04), total yield per plant (0.29), and mature red yield (0.31). All deep learning models (CNN, MLP, and RF) had higher prediction accuracy values compared to RRBLUP for mature green yield and plant height. The CNN and MLP showed a 6% (0.33 vs. 0.31) and 19% (0.37 vs. 0.31) merit, respectively, for mature red yield, relative to the RRBLUP model. In contrast, the RRBLUP model was more advantageous (mean of 0.74) for yield components such as ten pod weight showing 8% (RF; 0.68), 10% (MLP; 0.67), and 13% (CNN; 0.65) increase in accuracy compared to the deep learning models. The deep learning models and RRBLUP have similar accuracies for plant width (0.60). There was no model that was superior across all traits using the whole SNP marker dataset for predictions. However, it was observed that BRR had the highest mean accuracy for first pod date (0.77), mature green yield (0.06), and total yield per plant (0.33). The MLP model was the most superior for flowering time (0.76) and plant height (0.73). CNN had the highest average accuracy for mature red yield (0.37), whereas RRBLUP had the highest mean accuracy for ten pod weight (0.74). GBLUP was the most superior for predicting plant width (0.62).

Using an LD-based approach, marker pairs with LD coefficient, *r*^*2*^ > 0.25, were excluded for analyses resulting in 7,690 SNP markers (Additional File 3, Table S1) used in performing genomewide predictions (Table 1). Using a subset of markers resulted in a significant difference relative to using whole marker data for the MLP model for predicting total yield per plant (0.32 vs. 0.25) using Student *t*-test (*P* = 0.0006) (Additional File 2, Table S8). Likewise, a significant difference was observed in using subset of markers for first pod date for MLP (0.77 vs. 0.74; Student *t*-test, *P* = 0.0115). Significantly higher mean accuracy was also observed for mature green yield using subsets of markers for the BRR model (0.10 vs. 0.06; *P* = 0.045; Student *t*-test). Superior accuracies were also observed for ten pod weight for the RF model using a subset of loci (0.72) compared to using the whole marker data (0.68; *P* = 0.009; Student *t*-test) for genomewide selection. There were no significant differences (*P* > 0.05) across the models in terms of mean accuracy values for traits such as flowering time and plant width for using both the whole marker dataset and the subset of markers in performing genomic predictions. Overall, the average prediction accuracy values for the ridge regression and BLUP models (BRR, GBLUP, and RRBLUP) and the deep learning models (CNN, MLP, and RF) were similar across traits, except for mature red yield and plant height, where there were 3% (0.70 vs. 0.72) and 10% (0.30 vs. 0.33) gain using the latter models, respectively.

Genomic heritability (*h*^*2*^) values ranged between 0.0 and 0.80, with ten pod weight and plant height having the highest *h*^*2*^ values at 0.80 and 0.76, respectively. Yield-related traits were the least heritable, with *h*^*2*^ at 0.0 (mature green yield) and 0.10 (mature red yield and total yield per plant). Plant width (0.31), first pod date (0.26), and flowering time (0.28) had moderately low values for *h*^*2*^.

The BLUP and ridge regression models showed advantages for traits such as total yield per plant, ten pod weight, first pod date, and plant width over the deep learning models. There was a direct relationship between reported broad-sense heritability values, *H*^*2*^, for the traits and accuracy of genomic selection where a significant (*P* < 0.05) correlation using the whole marker data (Spearman rank correlation coefficient, *ρ =* 0.72; *P* = 0.0018) and the subset of markers (*ρ* = 0.71; *P* = 0.0022) across all traits was observed.

**Table 1:** Mean prediction accuracies across the different Bayesian ridge regression and BLUP and deep learning models for yield and agronomic traits in chile pepper using whole marker and a subset (s) of SNP loci for genomic selection

**Bayesian and BLUP models**									
**Trait** ^1^	**BRR**	**BRR_s**	**GBLUP**	**GBLUP_s**	**RRBLUP**	**RRBLUP_s**	**Mean (across traits)**	**Mean**	**Mean** (***s***)
FPD	0.77	0.77	0.77	0.77	0.77	0.77	0.77	0.77	0.77
FT	0.75	0.76	0.76	0.76	0.75	0.76	0.76	0.75	0.76
GRN	0.06	**0.10** ^**2**^	0.05	0.03	0.02	0.02	0.05	0.04	0.05
PHT	0.70	0.72	0.69	0.70	0.69	0.71	0.70	0.69	0.71
PWDTH	0.61	0.61	0.62	0.61	0.60	0.62	0.61	0.61	0.61
RED	0.31	0.33	0.29	0.30	0.31	0.29	0.31	0.30	0.31
TPW	0.68	0.69	0.71	0.71	0.74	0.72	0.71	0.71	0.71
TYP	0.33	0.32	0.31	0.32	0.29	0.31	0.31	0.31	0.32
**Deep learning models**									
	**CNN**	**CNN_s**	**MLP**	**MLP_s**	**RF**	**RF_s**	**Mean (across traits)**	**Mean**	**Mean (** ***s*** **)**
FPD	0.75	0.75	0.74	**0.77** ^**3**^	0.76	0.76	0.75	0.75	0.76
FT	0.75	0.76	0.76	0.76	0.75	0.75	0.76	0.75	0.76
GRN	0.02	0.03	0.05	0.05	0.04	0.07	0.04	0.04	0.05
PHT	0.72	0.72	0.73	0.73	0.72	0.72	0.72	0.72	0.72
PWDTH	0.61	0.60	0.60	0.60	0.60	0.60	0.60	0.60	0.60
RED	0.37	0.35	0.33	0.34	0.30	0.30	0.33	0.33	0.33
TPW	0.65	0.67	0.67	0.70	0.68	**0.72** ^**5**^	0.67	0.67	0.70
TYP	0.30	0.30	0.25	**0.32** ^**4**^	0.29	0.32	0.30	0.28	0.31

^1^*FPD*- First pod date (0.73); *FT*- Flowering time (0.73); *GRN*- Mature green yield (0.58); *PHT*- Plant height (0.61); *PWDTH*- Plant width (0.41); *RED*- Mature red yield (0.20); *TPW*- Ten pod weight (0.88); *TYP*- Total yield per plant (0.20). Values in parentheses are broad-sense heritability (*H*^*2*^) for the traits as reported by Lozada et al. [30].

^2^ Mean prediction accuracy significantly different with the accuracy for whole genome marker data at *P* < 0.05 (*P* = 0.045; Student *t*-test).

^3^ Mean prediction accuracy significantly different with the accuracy for whole genome marker data at *P* < 0. 05 (*P* = 0.0115; Student *t*-test).

^4^ Mean prediction accuracy significantly different with the accuracy for whole genome marker data at *P* < 0. 001 (*P* = 0.0006; Student *t*-test).

^5^ Mean prediction accuracy significantly different with the accuracy for whole genome marker data at *P* < 0. 05 (*P* = 0.009; Student *t*-test).

### Genomic estimated breeding values and response to selection

**Fig. 1:**
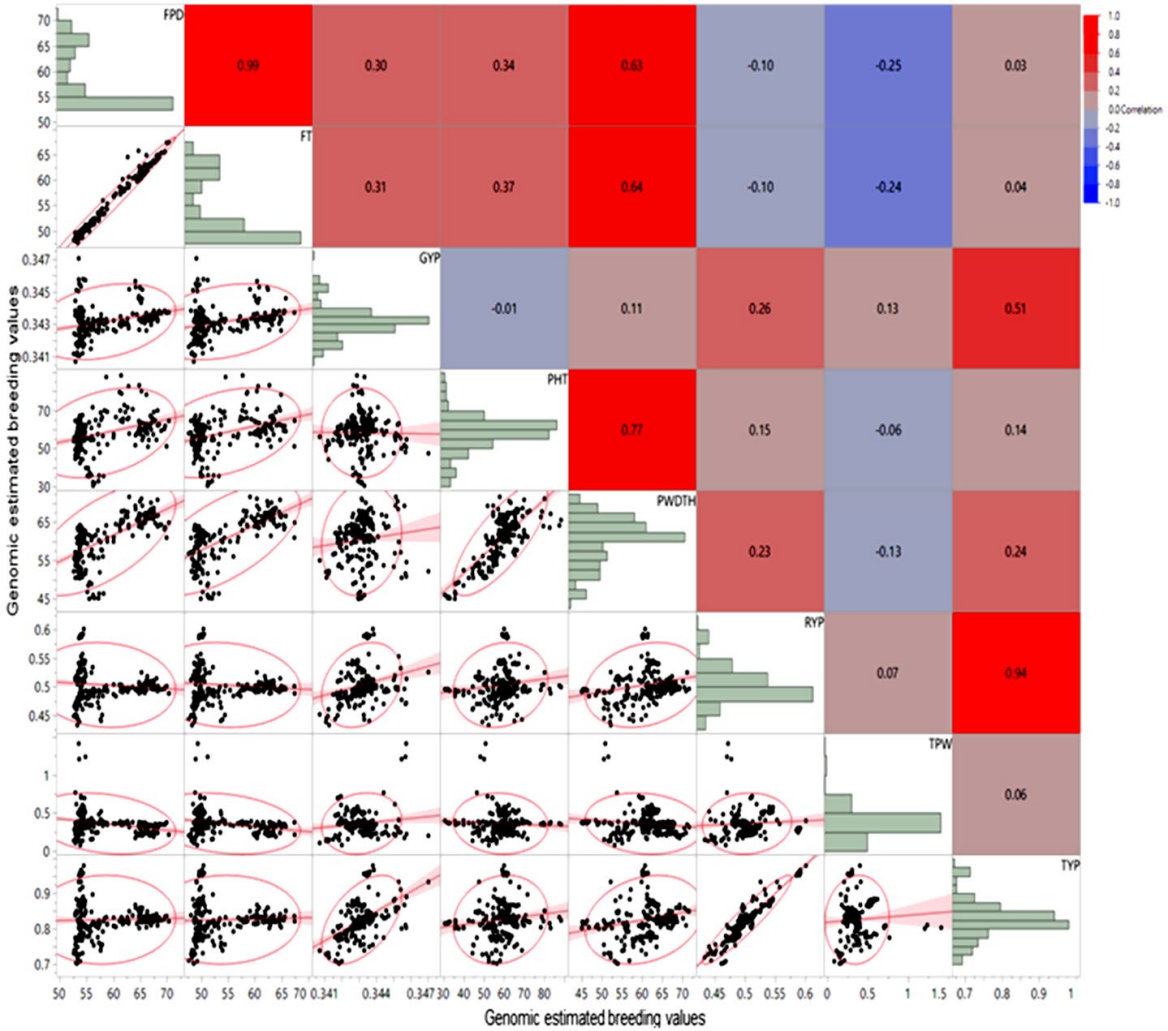
Correlation between genomic estimated breeding values for yield and agronomic traits in chile pepper using a Bayesian ridge regression genomewide prediction model. *FPD*- First pod date; *FT*- Flowering time; *GRN*- Mature green yield; *PHT*- Plant height; *PWDTH*- Plant width; *RED*- Mature red yield; *TPW*- Ten pod weight; *TYP*- Total yield per plant

Differences among the genomic estimated breeding values (GEBVs) were observed across the different models, where related traits showed higher values of correlation (Additional file 4, Table S1). For the BRR model using the whole marker dataset, for example, plant height and plant width GEBVs demonstrated highly significant correlation coefficient (*r* = 0.77; *P* < 0.0001) (Fig. 1). Similarly, first pod date and flowering time showed high correlation value (*r* = 0.99; *P* < 0.0001). Mature red and mature green yield had significant correlation with total yield per plant; however, ten pod weight did not have significant correlation with total yield per plant GEBVs. Across all genomic prediction models, marker datasets, and traits, GEBVs ranged between − 5.85E-08 (mature green) and 93.07 (plant height) (Table 2). Plant width had mean GEBVs of 30.81 (whole marker dataset) and 31.18 (LD-based marker dataset) across all prediction models, whereas flowering time had average values for GEBV of 27.59 and 27.68 for the whole marker and LD-based marker datasets, respectively. Skewed (non-normal) distributions for the GEBVs were observed across the traits (Shapiro Wilk test, *P* < 0.05) (Fig. 1).

**Table 2:** Summary statistics for the genomic estimated breeding values (GEBVs) across different prediction models for quantitative traits in chile pepper

Trait		Whole marker dataset					LD-based marker dataset				
	Genomic heritability (*h*^*2*^)	Mean	Std. dev	SE mean	Min	Max	Mean	Std. dev	SE mean	Min	Max
First pod date	0.26	29.69	29.04	0.83	-4.57	72.29	29.76	28.98	0.83	-5.07	70.54
Flowering time	0.28	27.59	27.22	0.78	-5.29	68.81	27.68	27.12	0.78	-5.29	66.93
Mature green yield	0.00	0.17	0.17	0.01	-5.85E-08	0.35	0.17	0.17	0.005	-0.0001	0.35
Mature red yield	0.10	0.26	0.25	0.01	-0.03	0.6	0.26	0.25	0.01	-0.04	0.6
Plant height	0.76	29.68	30.49	0.87	-29.01	95.66	29.35	30.69	0.88	-29.55	93.07
Plant width	0.31	30.81	30.31	0.87	-16.78	73.96	31.18	29.93	0.86	-16.26	72.66
Ten pod weight	0.80	0.19	0.24	0.01	-0.31	1.58	0.19	0.24	0.01	-0.29	1.56
Total yield per plant	0.10	0.43	0.4	0.01	-0.07	0.98	0.42	0.40	0.01	-0.08	0.96

The response to selection, *R*, for yield and yield components were evaluated by estimating selection differentials for various selection strategies, namely, phenotypic selection (PS) (selection using phenotypic (BLUP) values), genomic selection (GS) (selection using GEBV), and PS + GS (selection using both BLUP values and GEBV) and multiplying these selection differentials to the reported broad-sense heritability, *H*^*2*^, of the trait (Table 3). The values for *R* for green yield per plant were similar for PS, GS, and PS + GS. Using GEBVs exclusively for selecting the Top 20 selection candidates (i.e., 10% selection intensity) resulted in an overall decrease for the values for selection differential (*S*), and consequently, response to selection, for the other yield-related traits, viz. red yield per plant (-67%), ten pod weight (-3%), and total yield per plant (-33%), relative to PS. In contrast, selecting for the genotypes with the highest BLUP and highest GEBV (PS + GS) resulted in gain for *R* for red yield per plant (17%), ten pod weight (8%), and total yield per plant (33%). Using a PS + GS strategy, there were 11 (55%), 9 (45%), 17 (85%), and 9 (45%) individuals that were selected for mature green yield, mature red yield, ten pod weight, and total yield per plant, respectively.

**Table 3:** Response to selection (R) for total yield per plant and yield components using whole marker dataset for a Bayesian ridge regression model. The top 20 selection candidates (genotypes) with the highest phenotypic value (PV) and genomic estimated breeding values (GEBV), corresponding to a selection intensity of 10%, were selected for phenotypic selection (PS) and genomic selection (GS), respectively. The top 10% selection candidates with the highest PV and GEBV from PS and GS were selected in PS + GS.

Trait	*H* ^*2* 1^	Mean population without selection	Mean population with selection			Selection differential, *S*^2^			Response to selection, *R*^3^			Percent change relative to PS, *R*			No. of selection candidates selected (%)	
			PS	GS	PS + GS	PS	GS	PS + GS	PS	GS	PS + GS	PS	GS	PS + GS	PS + GS	
Mature green	0.58	0.34	0.35	0.35	0.36	0.01	0.01	0.02	0.01	0.01	0.01	-	0	0	11 (55%)	
Mature red	0.32	0.50	0.69	0.57	0.71	0.19	0.07	0.21	0.06	0.02	0.07	-	-67%	17%	9 (45%)	
Ten pod weight	0.88	0.36	0.77	0.76	0.8	0.41	0.4	0.44	0.36	0.35	0.39	-	-3%	8%	17 (85%)	
Total yield per plant	0.20	0.82	1.11	1.02	1.21	0.29	0.20	0.39	0.06	0.04	0.08	-	-33%	33%	9 (45%)	

^1^ Broad-sense heritability (*H*^*2*^) values reported by Lozada et al. [30]

^2^ Selection differential, *S = µ*_pop. with selection_ - *µ*_pop. without selection_

^3^ Response to selection, *R* = *H*^*2*^*S*

## Discussion

We present the first report of implementing ridge regression and machine learning models for the genomic prediction of complex traits in New Mexican chile peppers. Some advantages in terms of average accuracies for the deep learning models were observed for traits such as mature red yield and plant height, albeit was not consistent across the traits. The majority of the phenotypes did not show any advantage for the deep learning models, where the ridge regression and BLUP approaches showed higher average values for selection accuracy. The major reason of the lack of apparent or clear, strong merit or gain for the deep learning models could be the relatively small size of the population (*N* = 204) used for performing cross-validations and predictions, as the main requirement for using deep learning models is the quality and large size of the training data [31]. In chile peppers, the size of the training population was crucial to improve selection accuracy using deep learning models for morphometric fruit descriptors [8]. Consequently, to apply deep learning models in a chile pepper breeding program, a sufficiently large population size with sufficient genetic diversity is needed to train the prediction models. Phenotypic data collection for traits related to yield and yield components, nevertheless, has been a major limitation for the implementation of genomics-assisted breeding in the *Capsicum*, as majority of the cultivars remain hand-harvested, significantly impacting the throughput for phenotyping yield-related traits [2]. This phenotyping bottleneck can be circumvented by integrating robotics systems which can facilitate “human-like” harvesting in chile peppers, using high-throughput platforms to collect phenotypic data for yield-related traits, and ultimately developing machine harvestable cultivars [2, 32, 33]. While the implementation of mechanical-driven harvest in red chile peppers had been particularly more successful [34, 35], its application in New Mexican pod type green chile peppers is still at its inception. Recently, the ‘NuMex Odyssey’ pepper was developed, which demonstrates the potential of mechanical harvesting in green chile peppers [32]. For breeding populations with small *N*, ridge regression and BLUP models could be sufficient to provide desired prediction accuracy for complex traits in chile peppers, as shown in the current study. In other Solanaceous crops such as tomatoes (*Solanum lycopersicum*), using a small training population size of 96 was sufficient to achieve optimal accuracies for soluble solid content and fruit weight, where a GBLUP model showed significantly higher prediction accuracy compared to RF [12], possibly a consequence of using a small size of the training population.

The mean accuracy for using a marker subset from LD-based selection was observed to be higher for some traits, indicating that using a subset of loci can potentially improve the accuracy of prediction for complex traits in chile peppers. Notably, using subset of markers for deep learning models such as the MLP and RF resulted in increased accuracies for traits such as first pod date, ten pod weight, and total yield per plant, even under a relatively small size of the training population. Likewise, an increase in prediction accuracy was observed when using a subset of markers derived from LD-tagging for fruit length, fruit shape, fruit width, and pericarp thickness in peppers [19]. The exclusion of the effects of redundant SNPs could have resulted in increased accuracy for genomic prediction [8] for a number of traits in the current study. Varying levels of LD were previously observed across different populations of chile peppers indicating the influence of different factors such as population structure, recombination hotspots, and selective sweeps on the patterns of LD [36–39]. The extent of LD for this population has been reported at ~ 2.82 Mb [30], which can potentially explain why a lower number of markers was sufficient to capture the large LD blocks present, resulting in an overall improvement in prediction accuracy across several traits. For populations with larger extent of LD, lesser number of markers can be used for performing predictions, whereas for panels with rapid LD decay, more loci should be included in the prediction models [40]. Decreasing the number of markers for genomic selection can potentially improve computational power without compromising the accuracy achieved in performing predictions for several traits in chile peppers [8].

Heritability values tend to have a direct relationship with the accuracy of genomewide prediction, consistent with previous studies [13, 14, 41] indicating its major impact on implementing genomic prediction in plant improvement programs. In the present work, a moderately strong positive relationship was observed between heritability and prediction accuracy. Highly heritable traits such as first pod date, flowering time, plant height, and plant width, generally showed generally higher prediction accuracies, whereas lowly heritable traits such as yield and the yield components have lower observed prediction values. Similarly, high prediction performance was observed for highly heritable traits in pepper such as fruit size and shape [8]. Exception, however, was observed for the mature green yield (*H*^*2*^ = 0.58 [30]; *r* = 0.04 and 0.05) in the current study, which could be due to the prediction models not being able to fully capture the marker effects resulting in lower prediction accuracies for this trait. The genomic heritability for mature green yield was observed to be close to 0, demonstrating that minimal or no additive genetic effects were identified, consequently resulting in lower prediction accuracies, particularly for models that capture only this type of effects.

While it is ideal to achieve higher or improved prediction accuracies for different traits by exploiting the ideal marker number, prediction models, or heritability (genetic architecture), it should be noted that the success of implementing genomic prediction approaches in breeding programs does not rely exclusively in achieving high selection accuracy, but how breeders can utilize genomic breeding values from genomic prediction, among others, in performing more informed breeding and selection decisions for crop improvement [18, 42]. Some traits such as yield, by nature, are composite, and hence, are more difficult to predict than others [43], resulting in generally lower selection accuracies. Selecting parental lines for hybridizations for improvement of cultivars and development of mapping populations could be made on the basis of the GEBVs, as these represent the additive genetic variance i.e., the portion of total genetic variation that is inherited from the parents to the offspring [43]. Ultimately, genomic selection can be implemented to improve the choice of parents to either preserve genetic diversity or optimize crossbreeding among lines [44]. Caution should be exercised when using GEBVs solely in selection as some lines having high estimated breeding values can have low observed phenotypic values, as indicated by low prediction accuracies for some traits. Values for the response to selection, *R*, were highest for a PS + GS strategy, where selection candidates were selected based on having the highest phenotypic and genomic estimated breeding values. An overall (positive) gain for an integrated selection approach was achieved for three yield-related traits, indicating that combining different selection strategies can help improve selection response, even for traits with low heritability, such as total yield per plant. Our observations were consistent with previous results in winter wheat for yield-related traits [13], where *R* was improved by combining PS and GS strategies in choosing selection candidates. Ultimately, using phenotypic and genomic breeding values could render more opportunities to select candidates that have a high probability to perform well across locations and years relative to lines selected based on the phenotype alone [42].

Altogether, our results indicated that deep learning models can be integrated in chile pepper breeding programs’ genomic prediction pipelines provided that there is a sufficiently large training population to perform genomewide selection. It would be necessary, however, to establish the ideal population size when implementing genomic selection in chile peppers in the future. Both previous studies by Hong et al. [19] and Kim et al. [22] used a diverse panel of at least 350 genotypes to predict fruit-related traits and capsaicinoid content in chile peppers, respectively. Another study for fruit morphology traits in chile pepper used a smaller population size (i.e., 180 lines) for genomic prediction [8]. While it has been recently shown that deep learning methods have competitive genomic selection accuracies even with small- to medium-sized test populations [45], these approaches might still remain as additional models rather than substitutes for the standard ridge regression models for *Capsicum* breeding and improvement programs, as the latter models still showed higher accuracies than the deep learning models for some of the evaluated traits in the current study. More empirical studies using larger training populations of chile pepper should be conducted. Integrating high-throughput phenotyping data such as spectral reflectance indices with deep learning models also showed great potential of achieving optimal prediction accuracies [45], and hence its utility in chile pepper breeding programs should be explored further. Heritability plays a major role in achieving optimal prediction accuracies and using a subset of markers can potentially improve accuracy values for complex traits in chile peppers using the deep learning models under a large training data.

## Conclusions

This study was conducted to determine the accuracy of various genomic prediction models in New Mexican chile peppers. The potential of using ridge regression and deep learning strategies to predict complex traits was demonstrated. Different models behaved differently in terms of prediction accuracy. Heritability is a major factor affecting the accuracy of genomic prediction. Using subsets of markers can potentially improve accuracy using deep learning models even under a relatively small size of the training population. The effect of population structure and genetic relatedness between the training and validation populations on genomic selection accuracy for yield and related traits in *Capsicum* should be examined in future studies under a large training population, as these could potentially affect the stability of the results from cross-validations. The use of BLUEs in performing predictions should also be explored in future studies, as these values could complement BLUPs for genomic selection. Overall, genomic prediction can be integrated in modern chile pepper breeding programs for the genetic improvement of key traits. The results observed here for ridge regression and deep learning prediction models further demonstrate that the latter approaches are “supplement to the genomic selection toolbox rather than replacement” [46], and a large training data would be essential when implementing these models in chile pepper breeding programs.

## Materials and methods

### Chile pepper germplasm

The chile pepper population comprised of 204 diverse genotypes previously used for the genomewide association analysis for yield, phenology, and other agronomic traits [30]. Briefly, the population consisted of four cultivated (*Capsicum annuum* L. including *C. annuum* var. *glabriusculum* (chiltepins), *C. baccatum*, *C. chinense*, and *C. frutescens*) and one wild (*C. chacoense*) species of chile pepper (Additional file 1, Table S3). The *C. annuum* comprised of breeding lines and cultivars previously released by the New Mexico State University Chile Pepper Breeding Program including ‘NuMex Heritage Big Jim’, ‘NuMex Joe E. Parker’, ‘NuMex Sandia Select’, and ‘NuMex Vaquero’. The *C. baccatum* consisted of aji pepper types from South America, whereas the *C. chinense* comprised of the habaneros and the ‘Superhots’. *C. frutescens* comprised of ‘Zimbabwe Bird’ and ‘Siling Labuyo’. Seeds were sown at the Fabián García Science Center Greenhouse, Las Cruces, NM, and were maintained under standard greenhouse conditions for cultivating chile pepper [47]. Seedlings with 8–10 true leaves were transplanted ~ 90 days after sowing in raised beds 4.5 m (~ 15ft) in length at the Leyendecker Plant Science Research Center, Las Cruces, NM (CRU) and at the Los Lunas Agricultural Science Center, Los Lunas, NM (LUN), 320 kms (200 miles) North of CRU. The transplants on each plot were ~ 0.30 m (1 ft) apart from each other. The plants were cultivated under standard management practices including furrow irrigation for growing chile pepper in New Mexico [48]. The CRU location has a characteristic Belen clay loam class, whereas the LUN has a mixture of sandy clay loam (Gila) and Belen soil types. Transplanting was done in April and May for the CRU and the LUN location, respectively. A single hand harvest of pepper fruit samples from up to five individual plants per genotype was conducted from September through October 2021.

### Collection and analysis of phenotypic data and heritability

The population was evaluated for different yield, phenology, and plant morphology-related traits in two environments (CRU and LUN) in New Mexico, USA [30]. Yield traits included total yield per plant, mature green yield, mature red yield, and ten pod weight; flowering time and first pod date comprised the phenology traits, whereas plant morphology-related traits consisted of plant height and plant width. Total yield per plant was represented as the total mature red and green fruit weight (in kgs.) collected from up to five individual plants per genotype, divided by the number of plants. Mature green and red comprised of the fresh weight (in kgs.) from green and red mature fruits, respectively. Ten pod weight was the weight of five red and five green fruit samples that were chosen randomly. The flowering time and first pod date represented the days when the genotypes start to flower and develop fruits, respectively, subtracted from the day of transplanting. Plant height is the average measurement of up to five individual plants from the ground to the top of the canopy. Plant width represented the mean measurement of the widest point of the canopy for up to five individual plants. Both plant height and width were measured in cm. The adjusted phenotypic values (BLUP) were calculated using the ACBD-R program for combined analyses across locations (COM), as described previously [30] (Additional file 4, Table S2). Briefly, the BLUP model consisted of the mean effect, unreplicated genotype effect, block effect, replicated check effects, effects of the interactions between environment and genotypes, effect of block nested into the location, and the residual effect, which were all regarded as random [30]. Estimating the genotypic values of selection candidate across multiple environments, at the plot level, for subsequent parental selection and hybridization, and breeding, was of primary interest in the current study and hence the BLUP values were used in performing cross-validations.

Broad-sense heritability (*H*^*2*^) values for the traits across locations were calculated using the formula: *H*^*2*^ = *σ*^*2*^_*G*_ / (*σ*^*2*^_*G*_ + *σ*^*2*^_*GE/n*_ + *σ*^*2*^_*e/nr*_), where *σ*^*2*^_*G*_ and *σ*^*2*^_*e*_ represent the variances due to genotype and residual, respectively; *σ*^*2*^_*GE*_ correspond to the variance due to genotype-by-environment interaction; and *n* is the number of environments or locations and were previously reported in [30]. Genomic heritability (*h*^*2*^) was calculated using the ‘mmer’ function in the package ‘sommer’ in R [49] using the formula *h*^*2*^ = *σ*^*2*^_*A*_/(*σ*^*2*^_*A*_ + *σ*^*2*^_*e*_), where *σ*^*2*^_*A*_ is the variance due to additive genetic effects and *σ*^*2*^_*e*_ is the variance due to residual, derived from using the whole marker dataset.

### Genotyping using GBS-SNP markers

Genotyping-by-sequencing (GBS) was implemented for genomewide single nucleotide polymorphism (SNP) marker discovery of the chile pepper samples as previously described [36]. Leaf tissue from individual seedlings at 4–8 leaf-stage were sampled for DNA extraction. Isolation of DNA was performed using a Qiagen DNEasy kit from with minor modifications from fresh leaf tissue (~ 50 mg) through the University of Minnesota Genomics Center (UMGC) DNA extraction facility (https://genomics.umn.edu/service/dna-extraction). A single-enzyme (*ApeKI*) GBS protocol was performed for ~ 100 ng of DNA per sample at UMGC (https://genomics.umn.edu/services/gbs). A detailed description of the GBS method conducted is presented in Lozada et al. [30]. Briefly, single-end (1 × 100) sequencing was performed using the Illumina NovaSeq 6000 sequencer (Illumina, CA, USA) for fragments ~ 300–744 bp in size. Raw FASTQ files were demultiplexed using the ‘bcl2fastq’ software. Trimmomatic [50] was used to remove the adapter sequences at the 3’ end. The FASTQ files were aligned to the ‘Zunla-1’ reference genome [51] using the Burrows-Wheeler Aligner [52]. FreeBayes Bayesian identifier [53] was implemented for the join calling of variants across all samples. Genetic variants with genotype rates < 95% and minor allele frequency < 0.01, and samples with genotype rates < 50% were excluded in the genotype data. Variant call format was converted to HapMap using the TASSEL [54] software. Numeric format from HapMap was derived using the “Converter” function in the iPAT program [55]. After filtering and quality control excluding the unmapped SNP loci, a total of 14,922 SNP markers previously used for GWAS [30] was used to perform genomewide selection of quantitative traits in chile peppers.

### Genomic prediction models

The phenotypic dataset used to perform genomic prediction consisted of values for BLUPs derived from adjusting the phenotypes based on an augmented design, as described in Lozada et al. [30]. Ridge regression best linear unbiased prediction (RRBLUP) [56], genomic best linear unbiased prediction (GBLUP) [57], Bayesian Ridge regression (BRR); and deep learning approaches, namely, random forest (RF), multilayer perceptron (MLP), and convolutional neural network (CNN) models were used to evaluate prediction accuracy for yield and agronomic traits in chile peppers for tested lines in tested environments. The RRBLUP and GBLUP models were implemented in 10-fold cross-validation (CV) and 100 iterations, whereas the BRR were implemented in 1,000 iterations, 200 burn-ins, and 10-fold CV in iPAT using the ‘rrBLUP’ [56] and ‘BGLR’ packages [58], respectively. While GBLUP, RRBLUP, and BRR could all be regarded as parametric regressions, they have several differences. The RRBLUP and GBLUP are penalized approaches [56], whereas BRR is a Bayesian approach [11, 58]. Furthermore, these prediction models have varying assumptions on the effects of markers [10]. With these, a set of similar, yet different commonly used BLUP and ridge regression genomic selection models were compared based on their prediction accuracies for yield and agronomic traits in *Capsicum*.

The GBLUP, RRBLUP, and BRR genomic prediction models take the form ***y*** = *µ* + ***Z****a* + *e*, where *µ* is the mean; ***Z*** is the incidence matrix for the random effects (design matrix of individuals (for GBLUP) and the design for the markers (BRR and RRBLUP)); *a* is the marker effect (for BRR and RRBLUP, *a* ~ *N*(0,σ^2^_*a*_), where σ^2^_*a*_ is the variance of markers) and *a* is the genetic effect of the individuals (GEBVs) for GBLUP with *a* ~ *N*(0, Gσ^2^_*a*_), where ***G*** is the genomic relationship matrix of the tested materials and σ^2^_*a*_ is the genetic variance; and *e* is the residual. Both RRBLUP and GBLUP assume loci to have a common variance making the models appropriate for traits affected by a large number of genes with minor effects [56, 59], and hence are regarded to be equivalent [60]. Nevertheless, in GBLUP, the dimension of the model is reduced and GEBV can be calculated directly without performing many iterations thereby increasing computational power and efficiency [57]. In BRR, the Gaussian prior results in the shrinkage of estimate similar to that of a ridge regression (RR), where all effects are reduced to a similar extent, where the mean (*µ*_*β*_ = 0) and variance (*σ*_*β*_^2^) is *σ*_*β*_^2^ ~ *χ*^−2^[58].

Random forest (RF) includes collection of multiple trees created using a set of predictors and later average from these trees is used for the final prediction and this helps to “decorrelate” the results from multiple identical trees. The predictive model based on RF can be expressed as: $${\widehat{y}}_{i}=\frac{1}{B}\sum _{b=1}^{B}{T}_{b}\left({x}_{i}\right)$$, where $${\widehat{y}}_{i}$$is the predicted value of the individual with genotype $${x}_{i}$$; *B* is the number of bootstrap samples and *T* represents the total number of trees. The RF model is generally computationally less intensive relative to other models such as the convolutional neural networks, as each tree is independent of each other and can be trained on different nodes. The functioning of the RF model can be delineated into four primary steps: (1) Bootstrap sampling is employed to select an individual plant *i (*$${y}_{i}$$, $${x}_{i})$$with replacement. This sampled individual may appear multiple times or not at all within the bootstrap samples (*b* = 1, …, *B*); (2) Feature selection is conducted by randomly choosing a subset of input variables (*SNPj*, *j* = 1, …, *J*), considering the number of features (max features). The objective is to identify the optimal feature set that minimizes the loss function, typically measured as Mean Squared Error (MSE); (3) At each node, the dataset is split into two new subsets (child nodes) based on the genotype of *SNPj*; and (4) Steps 2 and 3 are reiterated for each node until a predefined minimum node size or the specified maximum depth is reached. The final predicted value for an individual with genotype $${x}_{i}$$ is computed as the average of the values predicted by the decision trees in the forest.

The crucial hyperparameters for training the RF model include the number of trees, number of features sampled for each iteration, importance attributed to each feature, and the maximum depth of the trees. To optimize these hyperparameters, we employed randomized and grid search cross-validation techniques. The specific combinations explored during grid search cross-validation, following the randomized search, included the number of trees (200, 300, 500, 1000), max features (auto, sqrt), and max depth (40, 60, 80, 100). Our analysis was conducted using the ‘Random Forest Regressor’ and ‘Scikit-Learn’ libraries in Python 3.7 [27, 61].

The Multilayer perceptron (MLP) is a prominent choice in genomic prediction studies, serving as a feed-forward neural network. Comprising an input layer, multiple hidden layers, and an output layer, the MLP architecture finds widespread application in deep learning. In the context of genomic selection model training, the first hidden layer’s output materializes through an intricate process of weighted averaging and nonlinear transformations applied to each input feature, accompanied by a bias term ‘*b*’. The representation of this initial layer’s output (denoted as *Z*_*1*_) unfolds as follows: *Z*_*1*_ = *b*_*0*_ *+ W*_*0*_$f$_*0*_(*x*). In this equation, *Z*_*1*_ signifies the output for the first layer, *b*_*0*_ embodies the bias specific to the first layer, which is estimated based on the remaining weights (*W*_*0*_), ‘*x*’ denotes the genetic profiles of individual samples, and ‘$f$_*0*_’ characterizes a nonlinear activation function. Remarkably, this model undergoes sequential training, where the output from neurons in the preceding layer serves as the input for the successive layer. The overarching model expression can be concisely summarized as: *Z*_*k*_ = *b*_k−1_ + *W*_k−1_$f$_k−1_(*x*). Here, ‘*Z*_*k*_’ collectively represents the output vector pertinent to GEBVs and the terminology employed in this equation has been previously defined.

Hyperparameter optimization leveraged the intrinsic capabilities of the ‘Keras’ function and engaged grid-search cross-validation (CV). This approach meticulously selects parameter configurations that minimize the mean square error (MSE), following the principles elucidated by Pedregosa et al. [61] and Cho and Hegde [62]. Notably, this hyperparameter tuning procedure encompassed the entire dataset, encompassing all evaluated traits across the population. The optimized hyperparameters span a diverse set of attributes, including the learning rate (constant, adaptive), activation function (relu, linear, tanh, identity, logistic), solver algorithms (lbfgs, sgd, adam), the count of hidden layers (1, 4, 6, 8, 10), the number of neurons within a fully connected network (10, 19, 38, 50, 62, 98, 112, 150), drop-out rates (0, 0.01, 0.1, 0.2), the quantity of filters (16, 32, 64, 128), and regularization techniques (L1 and L2). The grid search CV process allocated 80% of the training data to hyperparameter optimization, with the remaining data reserved for validation, achieved through the independent split function of ‘Keras’ [27]. Further insights into the hyperparameter optimization process and the libraries employed are available in prior publications [28, 63, 64].

The Convolutional Neural Network (CNN) serves as a specialized neural network model tailored for scenarios where specific patterns exist within the input data. The CNN architecture employed here encompassed a structured arrangement, comprising an input layer, two convolutional layers, two pooling layers, a dense layer, a flattened layer, two dropout layers, and an output layer. To delve into the convolutional operation, we defined it as an integral transformation, denoted as: $s\left(t\right)=\left(f*k\right)\left(t\right)={\sum }_{x}k\left(t-x\right)f\left(x\right)$. Here, ‘$k$’ represents the kernel, and convolution effectively transforms ‘$f$’ into ‘$s\left(t\right)$’. This operation occurs iteratively across an infinite number of replicas of ‘$f$’, each shifting over the kernel along the chromosome. Notably, the filters employed consider the linkage disequilibrium along the chromosome. The incorporation of max-pooling layers after each convolutional layer serves to address dimensionality reduction and imparts invariance to the filters regarding minor input variations. The pooling layers achieved this by aggregating the output from the preceding convolutional layer, utilizing methods such as minimum, mean, and maximum operations.

Activation functions and dropout mechanisms were strategically applied, following both convolutional and dense layers. The optimization of hyperparameters leverages the inherent capabilities of the ‘Keras’ function, with the aid of grid-search cross-validation [27, 61]. Key hyperparameters subject to optimization for the CNN architecture encompass the activation function, learning rate, batch size, filter configurations, number of epochs, and solver selection. Additionally, techniques such as regularization, dropout, and early stopping play a pivotal role in mitigating overfitting within the model. Specifically, a dropout rate of 0.20 was employed during hyperparameter optimization for both MLP and CNN, in accordance with the approach outlined by Srivastava et al. [65].

To determine the effects of marker number on the accuracy of genomic prediction, a subset of markers derived from an LD-based approach was used. Pairwise LD, *r*^*2*^, was calculated in PLINK [66] for markers within a 200-kb window where pairs of SNPs with *r*^*2*^ > 0.25 were excluded for analyses. Accuracy of genomic selection was represented as the Pearson correlation coefficient between the GEBVs and phenotypic BLUP. Mean prediction accuracies for the different models across different number of SNP marker sets were compared using Student’s *t*-test in JMP Pro 16.2 [67]. The average genomic selection accuracies across different models for each trait were reported for the whole genome marker data (*r*_*w*_) and the subset of LD-derived markers (*r*_*s*_). The GEBVs were calculated by fitting each of the prediction models under the scenarios mentioned above for the *r*_*w*_ and *r*_*s*_ datasets. Genomic relationship (kinship) matrix for the genotypes was calculated using the method of VanRaden [68] in GAPIT v.3. [69] (Additional File 5, Figure S1). The relationship between reported broad-sense heritability values and accuracy of genomic prediction was assessed using Spearman rank correlation coefficient (*ρ*).

### Response to selection

To evaluate the potential gains achieved for yield and agronomic traits through different selection approaches, the Response to selection, *R* was estimated. Values for *R* were calculated for different breeding strategies: phenotypic selection (PS), genomic selection (GS), and an integrated PS and GS (PS + GS) approach using a 10% selection intensity (i.e., selecting the top 20 genotypes) based on phenotypic (BLUP) values, genomic estimated breeding values (GEBVs), and both BLUP and GEBVs, respectively. The *R* was represented as the product between broad-sense heritability values reported by Lozada et al. [30], and selection differential which is the difference between the mean of phenotypic values with selection applied and the mean of the population without selection (*R* = *H*^*2*^*S*_(µ with selection − µ without selection)_) [13, 14]. Response was compared based on the percent change relative to selection using the BLUP values (PS) [13, 14].

### Electronic supplementary material

Below is the link to the electronic supplementary material.

Supplementary Material 1: Additional file 1. Table S1. Genotype data (numeric) of the 204 chile pepper genotypes for 14,922 single nucleotide polymorphism (SNP) markers used for genomic prediction. Table S2. Genotype data (hapmap) of the 204 chile pepper genotypes for 14,922 single nucleotide polymorphism (SNP) markers used for genomic prediction. Table S3. Chile pepper genotypes used to perform cross-validations and genomewide selection using ridge regression and deep learning prediction models.

Supplementary Material 2: Additional file 2. Table S1. Genomic prediction accuracy across different ridge regression and deep learning models for 10 iterations and 10-fold cross validations for subset of markers derived from linkage disequilibrium (LD) coefficients and 14,922 SNP loci for first pod date. Table S2. Genomic prediction accuracy across different ridge regression and deep learning models for 10 iterations and 10-fold cross validations for subset of markers derived from linkage disequilibrium (LD) coefficients and 14,922 SNP loci for flowering time. Table S3. Genomic prediction accuracy across different ridge regression and deep learning models for 10 iterations and 10-fold cross validations for subset of markers derived from linkage disequilibrium (LD) coefficients and 14,922 SNP loci for mature green yield. Table S4. Genomic prediction accuracy across different ridge regression and deep learning models for 10 iterations and 10-fold cross validations for subset of markers derived from linkage disequilibrium (LD) coefficients and 14,922 SNP loci for plant height. Table S5. Genomic prediction accuracy across different ridge regression and deep learning models for 10 iterations and 10-fold cross validations for subset of markers derived from linkage disequilibrium (LD) coefficients and 14,922 SNP loci for plant width. Table S6. Genomic prediction accuracy across different ridge regression and deep learning models for 10 iterations and 10-fold cross validations for subset of markers derived from linkage disequilibrium (LD) coefficients and 14,922 SNP loci for mature red yield. Table S7. Genomic prediction accuracy across different ridge regression and deep learning models for 10 iterations and 10-fold cross validations for subset of markers derived from linkage disequilibrium (LD) coefficients and 14,922 SNP loci for ten pod weight. Table S8. Genomic prediction accuracy across different ridge regression and deep learning models for 10 iterations and 10-fold cross validations for subset of markers derived from linkage disequilibrium (LD) coefficients and 14,922 SNP loci for total yield per plant.

Supplementary Material 3: Additional file 3. Table S1. Genotype data (numeric) of the 204 chile pepper genotypes for 7,690 linkage disequilibrium (LD)-derived single nucleotide polymorphism (SNP) markers used for genomic prediction.

Supplementary Material 4: Additional file 4. Table S1. Genomic estimated breeding values (GEBVs) of the 204 chile pepper (*Capsicum* spp.) genotypes used for cross-validations and predictions across different ridge regression and deep learning models. Table S2. Phenotypic trait data (represented as best linear unbiased prediction (BLUP) values) for different complex traits in chile pepper used for cross-validations and genomewide selection.

Supplementary Material 5: Additional file 5. Figure S1. Heat map of the genomic relationship (kinship) matrix of the 204 chile pepper (Capsicum spp.) genotypes derived from 14,922 SNP markers.

## Data Availability

The datasets generated and/or analyzed during the current study are available in the FigShare repository. **Additional File 1**: 10.6084/m9.figshare.23402360. **Additional File 2**: 10.6084/m9.figshare.23402828.v1. **Additional File 3**: 10.6084/m9.figshare.23402816.v1. **Additional File 4**: 10.6084/m9.figshare.23404196.v1. **Additional File 5**: 10.6084/m9.figshare.24168426.
