# Supplementary figures and images for: Ridge regression and deep learning models for genome-wide selection of complex traits in New Mexican Chile peppers
Source: BMC Genom Data. 2023 Dec 18;24:80. doi: 10.1186/s12863-023-01179-6 (PMC10726521; doi:10.1186/s12863-023-01179-6)

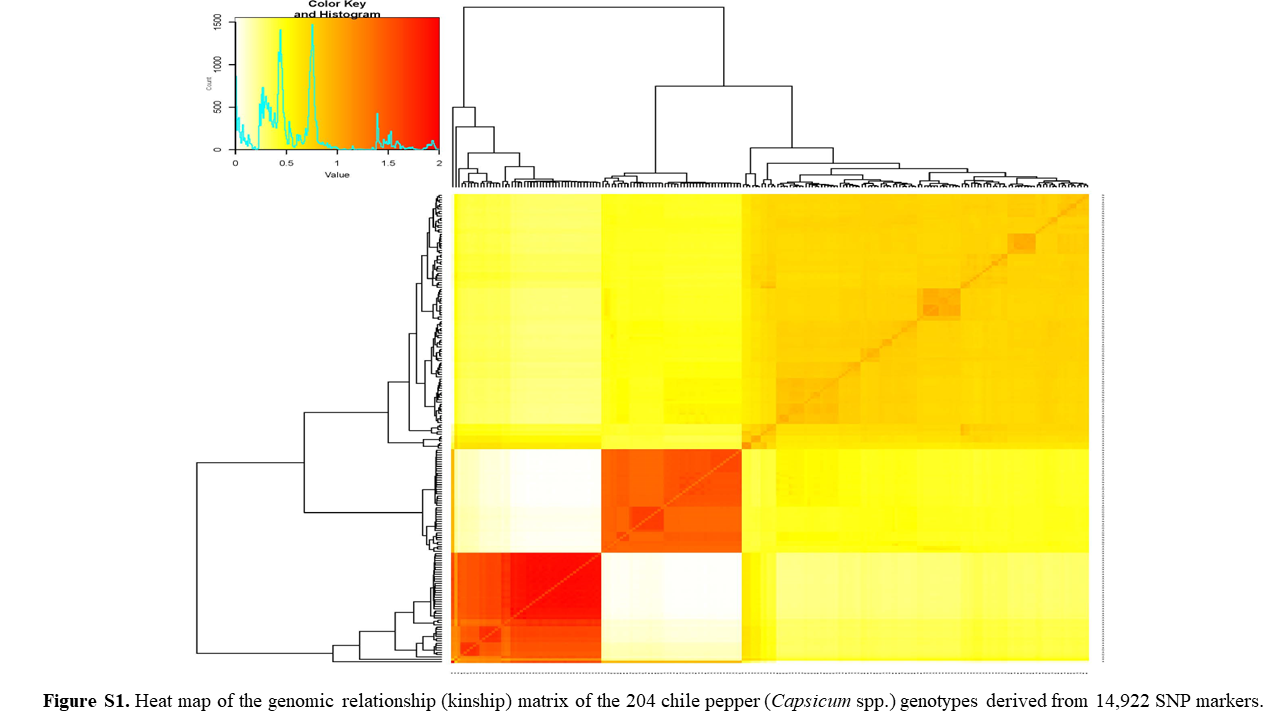

Supplement: Supplementary file 4 — Supplementary Material 4: Additional file 4. Table S1. Genomic estimated breeding values (GEBVs) of the 204 chile pepper (Capsicum spp.) genotypes used for cross-validations and predictions across different ridge regression and deep learning models. Table S2. Phenotypic trait data (represented as best linear unbiased prediction (BLUP) values) for different complex traits in chile pepper used for cross-validations and genomewide selection. [file 12863_2023_1179_MOESM4_ESM.png]
